# Fish bone ingestion mimicking aortic dissection: a case report

**DOI:** 10.1186/1757-1626-1-233

**Published:** 2008-10-10

**Authors:** Bjoern Kitzing, Yu Xuan Li

**Affiliations:** 1 Westmead Hospital, Cnr Hawkesbury and Darcy Roads, Sydney, New South Wales, Australia; 2 Royal Prince Alfred Hospital, Department of Radiology, Missenden Road, Sydney, New South Wales, Australia

## Abstract

**Introduction:**

The lodgement of fish bones during ingestion is a common complaint and can mimic other medical conditions such as aortic dissection.

**Case presentation:**

In this case a non-English speaking 63-year-old Chinese man presented to the emergency department with chest pain and hypotension. Subsequent spiral computed tomography angiography ruled out aortic dissection but demonstrated a lodged fish bone tenting the oesophagus in the non-contrast phase.

**Conclusion:**

This case highlights the importance of this imaging modality as well as the role of a good history in establishing an early diagnosis.

## Introduction

We present a case of fish bone ingestion mimicking aortic dissection and demonstrate the importance of good history taking as well as the significance of the non-contrast phase of spiral CT angiography.

## Case presentation

A non-English speaking 63-year-old Chinese man with a medical history significant for smoking presented to the emergency department complaining of chest pain. Symptoms had begun 2 hours prior to hospital admission, and consisted of a sharp, stabbing, substernal pain, starting shortly after a meal, and progressing gradually since then. The pain radiated to the patient's back, jaw and left arm. It was also pleuritic in nature as well as worse on swallowing and talking. The medical history was otherwise unremarkable.

On physical examination the patient appeared restless. His blood pressure was 116/86 on the right side and 96/56 on the left, pulse was 60 beats/min and regular, respiratory rate was 16 breaths/min and oral temperature was 36.8°C. His examination also revealed normal jugular venous pressure and normal breath sounds over both lung bases. The chest pain could not be reproduced on palpation. Heart sounds were distant, and peripheral pulses were normal. The remainder of the examination findings were unremarkable.

The ECG performed on hospital admission showed normal sinus rhythm with concave ST-elevation in V2 and V3. The initial creatinine phosphokinase concentration was 329 ng/mL (normal reference range, 30 to 220 ng/mL), the MB isoform fraction was under 3%, and troponin T concentration was under 10 ng/mL which is also within the normal reference range. The WBC count was 7700 cells/μL with 50% neutrophils.

**Figure 1:**
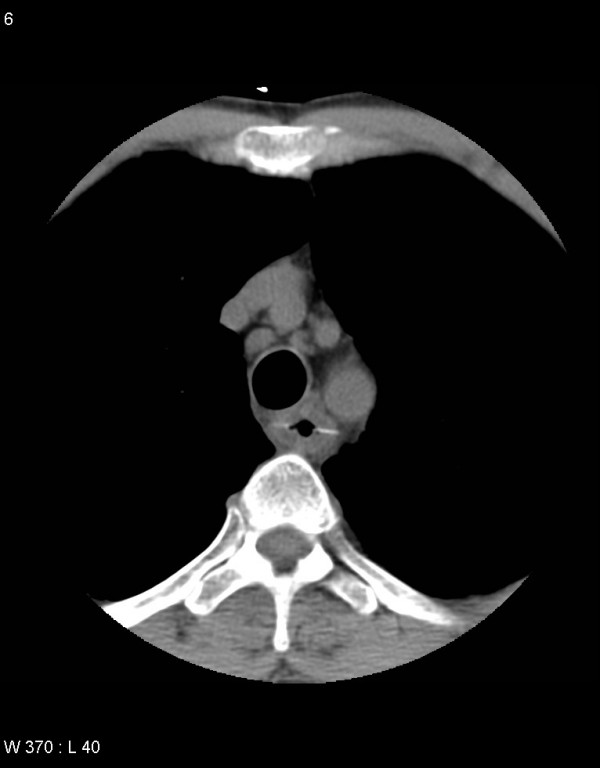
Select axial image of non-contrast phase CT showing a fish bone measuring 3 cm in the oesophagus.

**Figure 2:**
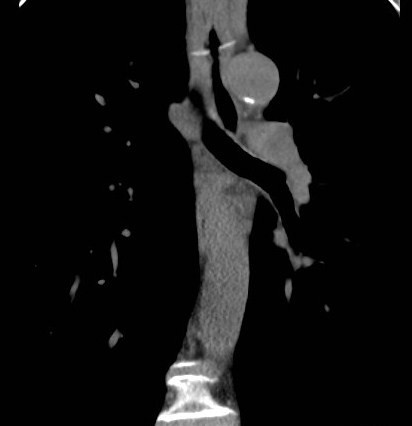
Select coronal image of non-contrast phase CT showing the fish bone lying transversely tenting the oesophagus.

The chest radiograph obtained showed no abnormalities. 4 hours after admission the patient became hypotensive (systolic blood pressure was 80) and bradycardic (50 beats/min). Due to concerns for an underlying aortic dissection, a CT spiral angiography (Fig. 1 and 2) was ordered.

The CT spiral angiography ruled out aortic dissection. However, multiple linear calcific densities were seen in the oesophagus and in the small bowel on the non-contrast phase of the study. One of the linear calcific densities was in a transverse orientation and seen tenting the oesophagus. The patient gave a history of ingesting fish half an hour prior to the onset of pain. The diagnosis of fish bone ingestion mimicking aortic dissection was made. Rigid endoscopy was performed and a fish bone was removed from the oesophagus approximately 20 cm from the incisor. No bleeding or ulceration was seen. Postoperative vitals were of a pulse of 72 beats/min and blood pressure of 125/70. Transthoracic echocardiogram showed that left and right ventricular function was normal, and that there was no apparent valvular disease. Serial troponin tests were negative. The patient had an uneventful recovery with intravenous antibiotic cover and was discharged home on Day 3 following admission.

## Conclusion

The lodgement of fish bones during ingestion is a frequent complaint. Its main complications are airway obstruction and oesophageal trauma. Rarer complications reported in the literature include the development of aortoesophageal fistula [1], or migration along an atypical tract. A careful history is important in establishing an early diagnosis. In this particular case, the patient was non-English speaking leading to difficulty in obtaining a satisfactory history.

The patient's presentation of chest pain and hypotension mimicked that of aortic dissection. Hypotension is often clinically associated with haemodynamic compromise and suggests major injury. However, the hypotension in this case was due to vagal plexus stimulation by the transversely orientated fish bone. The patient had bradycardia rather than tachycardia which supported the diagnosis of a vagal overtone rather than haemodynamic compromise.

The clinical appearance of aortic dissection is diverse. Patients with aortic dissection may have signs and symptoms that mimic myocardial infarction, aortic regurgitation, hypertensive crisis, pericarditis, cholecystitis, cerebrovascular accident, spinal cord ischaemia, mesenteric infarction, superior vena cava syndrome, acute pulmonary disorders, or mediastinal neoplasms [2]. Conversely, other diseases may look like aortic dissection [3,4].

Previously, the gold standard diagnosis of aortic dissection was formal angiography. Following the introduction of multi-detector computer tomography, the imaging modality of choice is spiral CT angiography. Whilst the contrast phase of the study which outlines the lumen is sensitive for dissection flap, the non-contrast phase of the study is sensitive for acute haematoma, intramural haematoma and aortic calcification. It is also useful in depicting other pathological entities with similar manifestations such as a lodged fish bone as in this case.

To conclude, this is a case of vagal hypotension due to lodged fish bone mimicking aortic dissection. The clinical pathway demonstrated the importance of good history taking and also the significance of the non-contrast phase of spiral CT angiography.

## Abbreviations

CT: computed tomography; ECG: electrocardiogram; WBC: white blood cell.

## Competing interests

The authors declare that they have no competing interests.

## Authors' contributions

BK made substantial contributions to conception and design and drafted the manuscript. YXL revised it critically for important intellectual content and gave final approval of the version to be published.

## Consent

Written informed consent was obtained from the patient for publication of this case report and all accompanying images. A copy of the written consent is available for review by the Editor-in-Chief of this journal.
